# TTQR: A Traffic- and Thermal-Aware Q-Routing for 3D Network-on-Chip

**DOI:** 10.3390/s22228721

**Published:** 2022-11-11

**Authors:** Hanyan Liu, Xiaowen Chen, Yunping Zhao, Chen Li, Jianzhuang Lu

**Affiliations:** The College of Computer Science, National University of Defense Technology, Changsha 410073, China

**Keywords:** 3D network-on-chip, adaptive routing algorithm, Q-learning, Q-routing

## Abstract

The die-stacking structure of 3D network-on-chips (3D NoC) leads to high power density and unequal thermal conductance between different layers, which results in low reliability and performance degradation of 3D NoCs. Congestion-aware adaptive routing, which is capable of balancing the network’s traffic load, can alleviate congestion and thermal problems so as to improve the performance of the network. In this study, we propose a traffic- and thermal-aware Q-routing algorithm (TTQR) based on Q-learning, a reinforcement learning method. The proposed algorithm saves the local traffic status and the global temperature information to the Q1-table and Q2-table, respectively. The values of two tables are updated by the packet header and saved in a small size, which saves the hardware overhead. Based on the ratio of the Q1-value to the Q2-value corresponding to each direction, the packet’s output port is selected. As a result, packets are transferred to the chosen path to alleviate thermal problems and achieve more balanced inter-layer traffic. Through the Access Noxim simulation platform, we compare the proposed routing algorithm with the TAAR routing algorithm. According to experimental results using synthetic traffic patterns, our proposed methods outperform the TAAR routing algorithm by an average of 63.6% and 41.4% in average latency and throughput, respectively.

## 1. Introduction

As technology scales and chip integration evolves, on-chip communication is becoming more crucial to system-on-chip (SoC) design. Network-on-chip (NoC) has become a communication infrastructure due to its scalability, reliability, and high throughput [1,2]. However, the performance improvement of 2D NoC is limited by the rapid decrease in packet latency due to the increase in physical distance between nodes. The 3D NoC enables smaller communication distances, larger bandwidth, and more flexible routing due to its 3D stacking architecture, which reduces latency and improves performance [3]. In the 3D NoC architecture, a processing element (PE) is connected to a router and propagates messages through the on-chip network to enable communication between PEs. The performance of NoC depends heavily on the routing algorithm, which determines the path of the packet through the network from the source node to the destination node. Routing algorithms are classified into three categories: deterministic, oblivious, and adaptive. Deterministic routing algorithms only provide a fixed route between a specified source and destination pair. Oblivious routing chooses a path randomly. Neither of the two above algorithms can reduce congestion because the routing decisions are made independently of the network conditions. However, traffic congestion must be minimized because it is the key factor limiting NoC’s performance [4].

Adaptive routing algorithms allow packets to choose among multiple alternative paths depending on the network conditions. The probability of network congestion can be effectively reduced by choosing an uncongested route to transmit packets. Generally, the adaptive routing algorithm can be divided into a routing function and a selection function. Firstly, a set of deadlock-free paths is provided by the routing function. Next, the selection function selects one of the paths from the set. The selection function is classified as either condition-independent or condition-aware [5].

Routing decisions in condition-independent algorithms, such as Zigzag [6] and random [7], are made regardless of network congestion. Load balancing may be affected because the network state is not taken into account in this strategy. On the contrary, condition-aware algorithms take the network’s congestion and thermal state into account during routing decisions. To avoid hotspots and alleviate contention [8,9,10], several approaches have been proposed. ANOC [9] is proposed to use cluster-based networks to reduce network congestion, which increases hardware costs. Many other condition-aware algorithms [8,10] also consider regional conditions. However, they are effective only when the cores communicate with other cores in the vicinity. An unequal distribution of traffic load may result from routing decisions based on local congestion information [11], since they cannot solve the global load balancing problem. Q-learning-based adaptive routing methods have been thoroughly investigated. Ref. Farahnakian et al. [12] proposed a Q-learning-based adaptive routing algorithm named Q-routing. A table must be kept on each router in order for Q-routing to work. Values in the Q-table indicate estimates of the time required from each output port to reach the destination node, which is used for selecting a path. Ref. Liu et al. [13] proposed QFCAR-W routing algorithm, which is inspired by the NoC fault-tolerant Q-learning technique.

The main contribution of this paper is to propose a 3D NoC adaptive routing method based on the Q-learning mechanism called the traffic- and thermal-aware Q-routing algorithm (TTQR). TTQR is an improved Q-routing algorithm that optimizes the overhead area of the traditional Q-routing algorithm from the aspects of cancellation of dedicated links and simplification of the Q-table. Moreover, the single-objective optimization is changed into a multi-objective optimization algorithm. There are two table in each router. The Q1-table provides the buffer status of the neighboring node while the Q2-table provides global thermal information, which is updated based on the average temperature information from the header of the received packets. The TTQR approach can estimate and predict the congestion and temperature conditions of the network and use this information for routing decisions to select a less congested path or an area with lower temperatures. According to experimental results using synthetic traffic patterns, our proposed methods outperform the TAAR routing algorithm by an average of 63.6% and 41.4% in average latency and throughput, respectively.

The rest of this article is organized as follows: in Section 2, we review and discuss the development of related routing algorithms; Section 3 presents the basic knowledge of Q-learning and Q-routing; the proposed TTQR method is presented in Section 4; The simulation results are displayed and discussed in Section 5; and Section 6 is the conclusion of this study.

## 2. Related Work

A large number of traffic- and thermal-aware routing algorithms have been proposed to address the challenges of uneven temperature distribution and traffic congestion in 3D NoC. The main differences among them are the routing direction selection and the run-time thermal management (RTM) approach. Path selection is based on the set of deadlock-free routing directions at each routing node, from which an optimal direction is chosen to reduce congestion. According to the RTM technique, there are two categories for 3D NoC routing algorithms: reactive thermal management [10,14,15] and proactive thermal management [16,17,18].

### 2.1. Reactive Techniques

The main feature of this type of approach is how to make a routing decision to change the traffic load distribution when a dormant router has already occurred. TLAR [14] is a topology-based routing algorithm that routes vertically and horizontally separately to achieve traffic load balancing between layers. TLAR achieves thermal balancing between horizontal routing layers by employing a combination of deterministic and adaptive routing algorithms. However, TLAR primarily performs downward routing when there are insufficient horizontal routing directions in the non-stationary irregular mesh. As a result, there is a traffic congestion problem in the lowest layer, and the network latency increases rapidly. TAAR [10] is a cascading routing algorithm that uses a topology table to collect throttling information to bypass the throttled router. TTABR [15] enables packets to bypass congested areas by dynamically selecting a minimum path or a wrap-around path. The wrap-around path, i.e., non-minimal path, alleviates the over-contention problem in the minimal path region. QTTAR [19] learns the network state through a Q-learning algorithm to deliver packets to low-temperature and low-blocking regions. In this algorithm, the first step collects all deadlock-free routing directions according to throttled information in the smallest region, and the second step selects the optimal routing direction. Ref. Taheri et al. [20] proposed a reactive routing algorithm for dynamically bypassing packets from hot regions with throttled nodes. In order to offer path variety for packet transmission at lateral routing, the method suggests two virtual channels. Path diversity enables the dispersing of the distribution of heat to reduce the maximum temperature.

### 2.2. Proactive Techniques

The main feature of proactive techniques is that researchers actively adjust the traffic load distribution of the network before the routers are limited to prevent localized regions from overheating and causing the routers to go dormant. In some proactive approaches [16,17], the routing strategy relies on the structural properties of the 3D on-chip network, i.e., the farther the router is from the heat sink, the more its ability to dissipate heat is lessened. In order to bypass the throttled nodes, ref. Chao et al. [16] proposed a downward routing technique to route packets to the lowest layer. Nevertheless, downward routing causes wasteful traffic movement and increases the overhead delay of message transmission. Some methods [18,21], on the other hand, present a thermal model to predict the temperature of the networks with the intention of changing the traffic loading of the router before it reaches the temperature threshold. For example, PTB3R [21] proposes a thermal metric called mean time to throttle (MTTT) for thermal budgeting. Authors in PTDBA [22] regulate the size of the router’s input buffers to balance the distribution of heat. However, because PTB3R and PTDBA’s predictions are based on data from past traffic loads, they are unable to accurately reflect the network’s current situation. Sivakumar et al. [23] propose a stochastic-based genetic algorithm to shorten the guided routing path length by replacing the location of TSVs. Cao et al. [24] publishes a comprehensive assessment of optimization methods for thermal problems in 3D NoC, most of which are active types.

## 3. Preliminaries

### 3.1. Q-Learning

In recent years, artificial intelligence (AI) [25,26,27,28] has gained a lot of ground in various engineering fields. Reinforcement learning [29] is a technique for obtaining the best choice when the system offers multiple choices. It has received a lot of attention in the past since it offers a practical answer to issues for which analytically definable optimal solutions are either unavailable or difficult to obtain. The learning strategy is founded on the rationale that, if a behavior is followed by a positive condition or development, the inclination to repeat that activity will be increased. On the other hand, if the situation deteriorates, the specific behavior should be penalized as necessary.

Q-learning [30] is one of the methods of reinforcement learning. In the Q-learning methodology, the learning agent first investigates an online environment model and uses this information to identify an efficient control strategy for a particular task. A Q-value is the expectation of the benefit that can be obtained by taking action *a* at a given moment in a given state *s*. The goal of the Q-Learning approach to learning is to build a Q-table with state as the row data and action as the column data. The Q-value in the Q-Table is constantly updated by the reward brought by each action, which is used as a basis to judge the next action.

### 3.2. Q-Routing

The Q-routing is an adaptive routing algorithm based on the Q-learning model proposed by Farahnakian et al. [12], which was first applied to solve the problem of network congestion.

The main idea of Q-routing is to store a Q-table at each router to evaluate the quality of the alternative paths. The Q-table stores an estimate of the time taken from each output port to the destination node. The size of the Q-table is Nnode×Nport, where Nnode represents the number of nodes in the NoC and Nport represents the total number of router output ports. Every time a router sends a packet to one of its neighbors, the value of the Q-table is updated based on the basic update mechanism. Once the packet has been successfully transmitted to the downstream node, the downstream router generates a learning packet with local and global traffic information and then returns it to the upstream neighboring node via a dedicated link. This method allows a node to gradually add additional global information to its Q-value as it routes data packets. The purpose of Q-routing is to learn and predict the state of the NoC as closely as possible to the real situation. By consulting the Q-table, we can choose paths with less contention when making routing decisions. Q-values are updated by the following rule:

(1) $$Q\left[s,a\right]\leftarrow Q\left[s,a\right]+\alpha (r+\gamma \cdot \max_{\alpha^{\prime }}Q\left[s^{\prime },a^{\prime }\right]-Q\left[s,a\right])$$

In this equation, the reward for switching from state *s* to state $s^{\prime }$ is *r*. $\max Q[s^{\prime },s^{\prime }]$ is the highest payment for estimating future states. α is the learning rate (0<α<1), which controls how fast the agent adapts to environmental changes.

Table 1 shows the regular Q-table of nodes in a two-dimensional network with a 3×3 mesh topology. Each row in the Q-table represents a destination. In 3D NoC, there are several times as many nodes as there are layers. Hence, the area overhead of the Q-table is increased because each destination in the network has its own row.

## 4. Traffic- and Thermal-Aware Q-Routing Algorithm (TTQR)

Our proposed method is able to balance the network traffic burden by selecting relatively idle output ports or routing directions pointing to low-temperature regions. Inspired by the literature [31,32], we propose a multi-objective decision-making approach. The proposed technique employs both traffic and temperature as information for Q-routing and is therefore called traffic- and thermal-aware Q-routing (TTQR). For analysis, we divide the routing algorithm into routing functions and selection functions. The first step in implementing TTQR is to collect a set of available deadlock-free channel ports through the routing function. The number of elements in the set is determined by the position of the current node and the destination node. In the second step, we propose a selection function based on Q-learning. The form and content of two Q-tables and their update rules are introduced. Finally, a pseudo-code is used to illustrate the routing method in detail.

### 4.1. Routing Function

**Figure 1:**
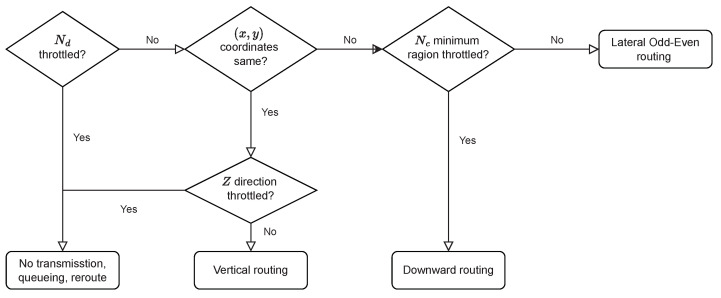
Algorithm flow for the routing function of a traffic- and thermal-aware Q-routing (TTQR).

The first step of TTQR is to identify throttled nodes in the minimum area of the current node and then select a set of deadlock-free routing directions. When the routing function is deterministic, the flexibility of routable directions diminishes rapidly when the minimum region is flooded by throttled nodes. As a result, there are only a few deadlock-free directions to pick from during routing decision-making. In order to provide alternative deadlock-free paths, we adopt the odd–even turn model of horizontal routing, whereas the inter-layer routing is based on a downward scheme. The deadlock-free paths are selected using intra- or inter-layer routing, independently configured to prevent circular dependencies between horizontal and vertical directions. Figure 1 depicts the flowchart of the routing function, where $N_{s}$ and $N_{d}$ denote the current node and the destination node, respectively. When the destination node is not throttled, it determines whether the *x* and *y* coordinate pairs of the current node are consistent with those of the destination. If they are consistent, the routability of the vertical path is determined. If not, the route direction is divided into horizontal directions and downward routing. In this case, if there are no throttled nodes in the minimum region, the routable directions are initially selected by the routing function to ensure path diversity. Once throttled nodes are detected in the minimum region, downward routing occurs between layers. Through this process, TTQR finds the routes by bypassing the area containing the hotspots.

The majority of heat-aware routing algorithms in use nowadays adopt a downward scheme, but the lateral plane still departs from routable paths, creating an overabundance of traffic concentration in the bottom layers [15]. Therefore, TTQR employs adaptive routing when $N_{c}$ lies above the throttled layer to improve the flexibility of the routing path. When finding all possible paths in the region within the layer, the computational complexity is $O(4^{N})$, which significantly raises the overhead of the selection process [14]. Searching only the minimum region lowers the computational overhead.

**Figure 2:**
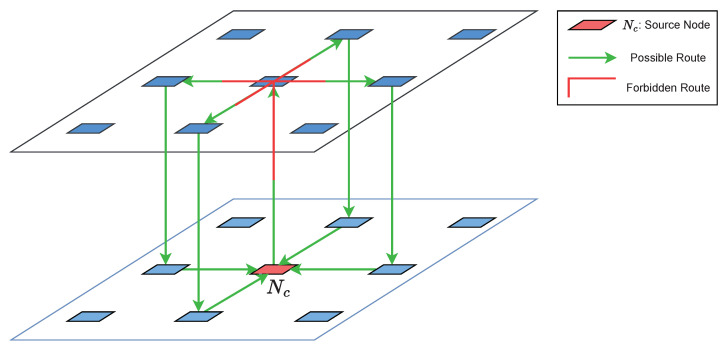
Deadlock-free routing mechanism.

TTQR prohibits upward routing followed by lateral routing to prevent circular dependencies between intra- and inter-layers. Only when the x and y coordinate pairs of $N_{c}$ and $N_{d}$ are equivalent, and the *z* coordinate of $N_{d}$ is smaller than that of $N_{c}$, is upward directional routing performed. This makes sure that there is no deadlock in the direction selected in the first step of TTQR, as shown in Figure 2.

### 4.2. Selection Function

The purpose of this step is to choose the direction to a non-congested port or low-temperature region based on the Q-table. Previous studies [12,33] have introduced Q-tables, where the path estimates for all nodes in the system are stored in each router. To minimize the size of the Q-table, instead of using the total number of nodes, we simplify the row index to the four directions of the inner layer. In a 3D NoC with a n×m×l mesh topology, the row size of the Q-table is reduced from n×m×l to 4. Each router keeps two Q-tables containing four directions and four Q-value fields.

#### 4.2.1. Q1-Table for Optimizing Latency

The value of the Q1-table is described as information about the throttling and input buffers of various nodes around the router. The value of Q1-table is the medium of traffic status in this direction, while a higher value indicates that the route in this direction is optional. Table 2 shows a Q1-table with fictitious values.

For example, the current nodes of Ne and Ns are the east neighbors of Nc. In the Q1-table, the value of the east row represents the contention estimate for the eastward transmission of the packet. The sum of the buffer free slots of a set of optional routing directions from the Ne node to the destination is the Q1-value of the east row.

After obtaining the estimated values for each direction, $N_{c}$ combines the old values with the estimated values to calculate the new values for the next routing decision. This is expressed in the Equation (2) as follows:

(2) $$Q1_{\left(b,z\right)}^{\prime }=Q1_{\left(b,z\right)}+\Delta Q1_{\left(b,z\right)},\;z\in \left\{\mathrm{north},\mathrm{south},\mathrm{east},\mathrm{west}\right\}$$

where $Q1_{\left(b,z\right)}$ denotes the old estimates that route from the current node to the *z*-direction, and $Q1_{\left(b,z\right)}^{\prime }$ is the new estimates updated by the message to be routed, calculated by $Q1_{\left(b,z\right)}$ and $\Delta Q1_{\left(b,z\right)}$. In this scenario, $Q1_{\left(b,z\right)}^{\prime }$ has a higher positive value when the Q1-value is larger. The parameter $\Delta Q1_{\left(b,z\right)}$ is the corrected incremental estimate, as expressed in Equation (3).

(3) $$\Delta Q1_{\left(b,z\right)}=\alpha \cdot \left(\sum S_{\mathrm{neighbor},y}-Q1_{\left(b,z\right)}\right),\;y\in \left\{\mathrm{north},\mathrm{south},\mathrm{east},\mathrm{west},\mathrm{up},\mathrm{down}\right\}$$

The $S_{neighbor,y}$ in Equation (3) denotes the total of the newly received estimates from neighboring nodes in routable path, i.e., it represents the fraction of the neighboring nodes’ input buffer states. $\Delta Q1_{\left(b,z\right)}$ acts as a corrector to update the new Q1-value using the old Q1-value and the estimate from the neighbor. The parameter α is the learning rate that determines the weights of the delta estimates. α is a value between 0 and 1, which determines the size of the old values to be covered when new information is updated to the Q1-table. When the learning rate is 0.6, we can obtain the best average delay (as determined by empirical trials).

#### 4.2.2. Q2-Table for Optimizing Temperature

Q2-table represents the estimated value of the average temperature of all intermediate routers from the current node to the destination node. All Q2-values are initially set to the outside temperature. A Q2-table with fictitious temperatures is shown in Table 3.

Our method differs from the traditional Q2-value update mechanism. Each router stores the average temperature of the nodes it passes through in the header of the message before sending the message to the next router. When a router receives a packet, it calculates the new Q2-value relative to that direction using the average temperature from the packet’s header. Actually, the new Q2-value is an estimate of the average temperature of the nodes that packets from that direction pass through. This information is useful for packets going in the opposite direction of the current packet. Therefore, the learning packets are skipped. As a result, no additional dedicated link is required to transmit the learning packet.

Assume the router *r* receives the packet *p* through the input port *i*. The router *r* will extract temperature information (Avg_Temp) from the packet header, which represents the average temperature of the router through which the packet *p* passed on its way from the source node to the current node. The router then uses Avg_Temp to calculate the Q2-value associated with direction *i*. Therefore, as packets leave the router *r* from the output *i*, they may experience this predicted temperature.

(4) Q2[i]=Q2[i]+α(Avg_Temp−Q2[i])

In addition, in order to update the Q2-table of the subsequent router, the current router must add its own temperature to Avg_Temp before transmitting the message. Avg_Temp is updated as follows:

(5) $$\begin{array}{cc} \mathrm{Avg}\_\mathrm{Temp}(\mathrm{new}) & =\left(\frac{\mathrm{Hop}\_\mathrm{Count}-1}{\mathrm{Hop}\_\mathrm{Count}}\right)\times \mathrm{Avg}\_\mathrm{Temp}(\mathrm{old})\\ \; & +\frac{1}{\mathrm{Hop}\_\mathrm{Count}}\times \mathrm{Local}\_\mathrm{Temp} \end{array}$$

where Hop_Count indicates the number of intermediate routers the packet has passed through, Avg_Temp(old) indicates the average temperature sensed by the packet in its previous state, and Local_Temp indicates the current router’s temperature.

### 4.3. Summary of TTQR

Assume a packet *p* is sent from the source *s* to the destination *d*. If router *r* receives the message, it determines that there is a throttled node in the minimum area and, if so, transmits the message to the next level. If not, the set of output channels in the horizontal plane is selected using parity turn routing, through which the message can be delivered to the node corresponding to the current layer node *c*. If the set species has only one output channel, then it is transmitted directly. If there are two output channels, the router chooses between them using its two Q1-values. The direction that has a greater ratio of Q1-value to Q2-value is chosen as the best output channel.

The pseudo-code for the TTQR routing algorithm is shown in Algorithm 1.
**Algorithm 1:** Sequential of the TTQR routing algorithm.**Input:** 
Q1-table (Q1), Q2-table (Q2), Routable Direction Set (DS), Source Node ($N_{s}$), Destination Node ($N_{d}$)**Output:** 
Action (*A*)                  ▹ The selection of the output port1:**if**$N_{s}=N_{d}$**then** return;                   ▹ Arrive to the destination2:**else if** Size(DS) = 1 **then** A=direction13:**else if** Q1(direction1)/Q2(direction1)=Q1(direction2)/Q2(direction2) **then**  A=random(direction1,direction2)4:**else if** Q1(direction1)/Q2(direction1)>Q1(direction2)/Q2(direction2) **then**  A=direction15:**else**A=direction26:**end if**

## 5. Simulation Results and Discussion

### 5.1. Simulation Setup

Simulations were performed through a cycle-accurate traffic-thermal co-simulation platform called Access Noxim [34], which integrates the NoC simulator Noxim and the architecture-level thermal model Hotspot. The simulation parameters for the network’s co-simulation are shown in Table 4. The 3D NoC is an 8×8×4 mesh structured network containing 256 tiles. We evaluated the average latency, throughput, temperature, and traffic load distribution of our proposed method and compared them with the TAAR routing algorithm. In this study, ablation studies were performed to analyze not only the performance of TAAR and TTQR in different traffic patterns, but also the performance of routing using the Q1-table only and the Q2-table only.

### 5.2. Analysis of Network Performance

**Figure 3:**
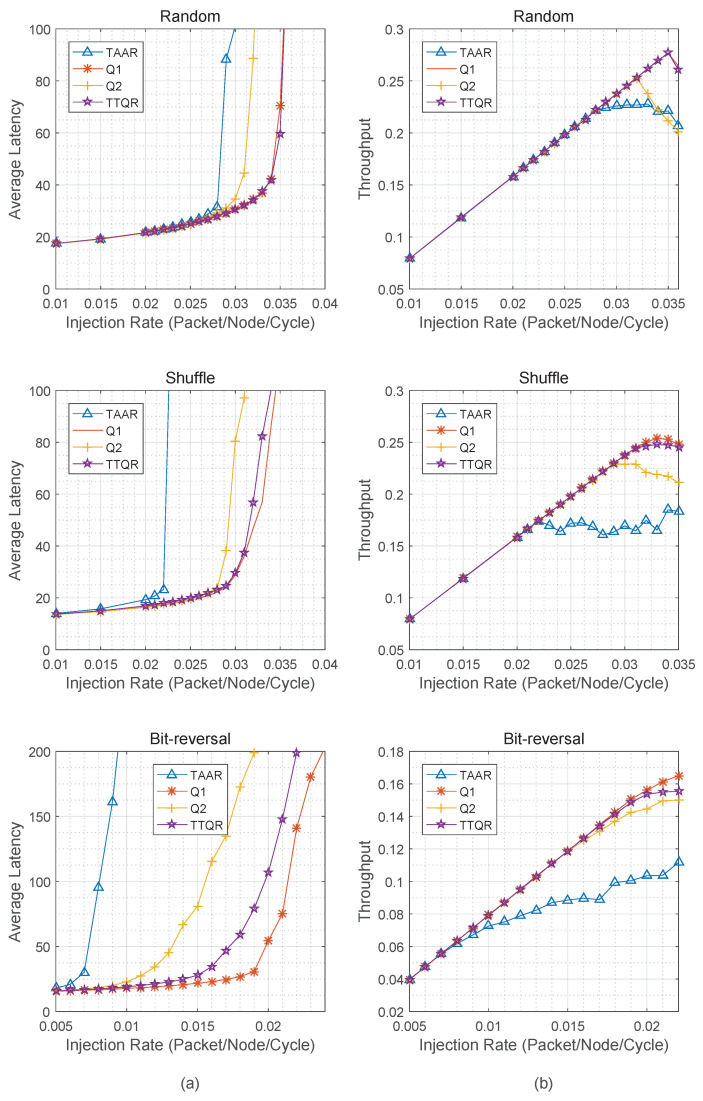
(**a**) Average latency of TTQR versus Q1, Q2, and TAAR. (**b**) Throughput of TTQR versus Q1, Q2, and TAAR.

We usually analyze the performance of NoC by measuring the average delay and throughput of packets under different injection rates. Figure 3a shows the average packet delays of TAAR, Q1, Q2, and TTQR under three different synthetic traffic patterns. It can be seen that the network packet delay increases exponentially with the injection rate. Regardless of the traffic pattern, the packet delay of TTQR is shorter than that of TAAR. This is because TAAR has periodic reconfiguration phases for topology table updates and routability checks for incoming packets. The Q-tables, on the other hand, are updated concurrently with the network’s daily operations in the TTQR routing algorithm. In addition, TAAR adopts the “store and forward” strategy and cascade routing, which leads to a longer waiting time for data packets. The results show that the network latency of TTQR is improved by 21%, 40.9%, and 128.6%, respectively, over TAAR. Figure 3b shows a comparison of the network throughput of TAAR, Q1, Q2, and TTQR under four different synthetic traffic patterns. Compared with TAAR, the throughput of TTQR is improved by 25.3–50.0%.

### 5.3. Analysis of Statistical Traffic Load Distribution (STLD)

**Figure 4:**
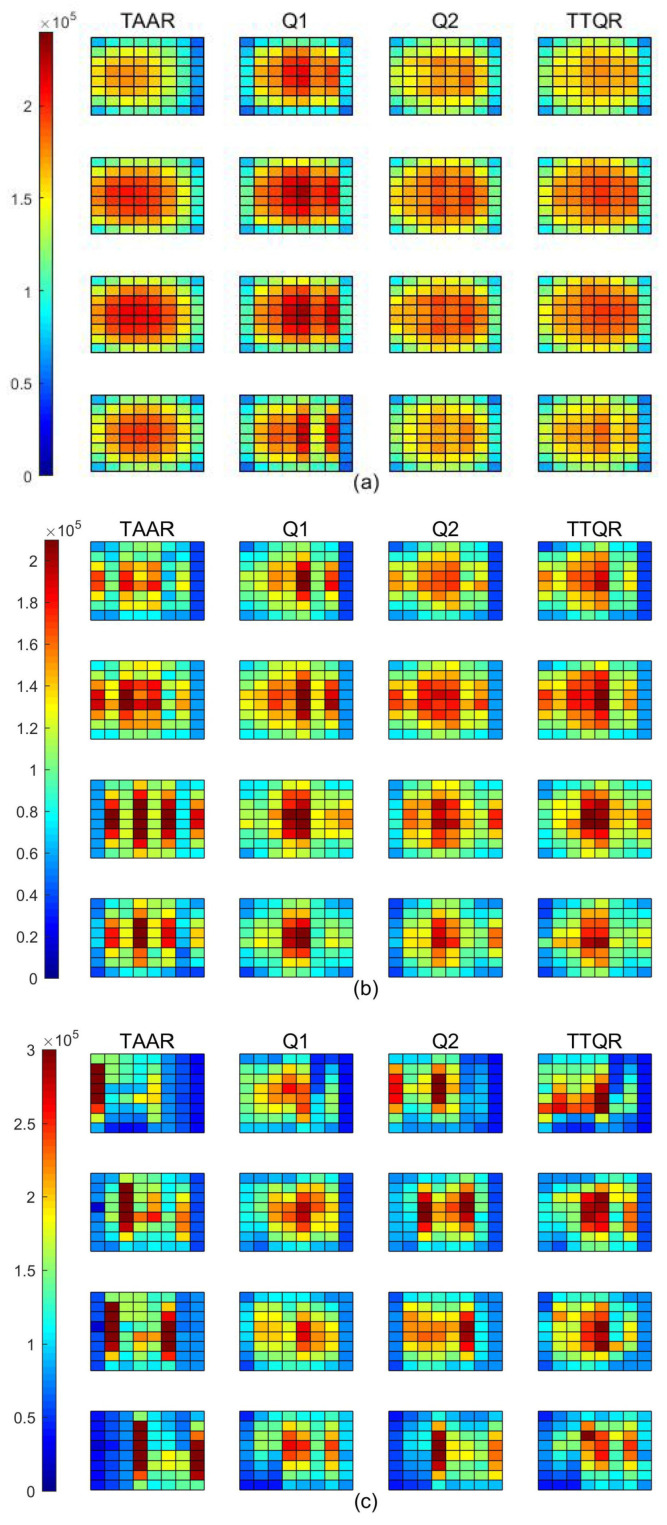
STLD under different traffic patterns. (**a**) Random. (**b**) Shuffle. (**c**) Bit-reversal.

Figure 4 shows the traffic load distribution (STLD) of the network in three synthetic traffic patterns. As indicated, the bottom layer’s traffic of TTQR is slightly denser than that of TTQR. This is because TAAR performs inter-layer routing without taking into account the traffic load information between the layers after confirming the intra-layer adaptability. The downward route is thus the first option when TAAR hits a throttled area and the target node is not in the same layer, leading to an imbalanced traffic load distribution between levels. Instead, we propose that the routing algorithm gradually directs traffic to the peripheral path, followed by downward routing, in the early stages of router overheating. Moreover, compared with TAAR, TTQR has a more balanced traffic distribution between layers.

### 5.4. Analysis of Temperature Distribution

**Figure 5:**
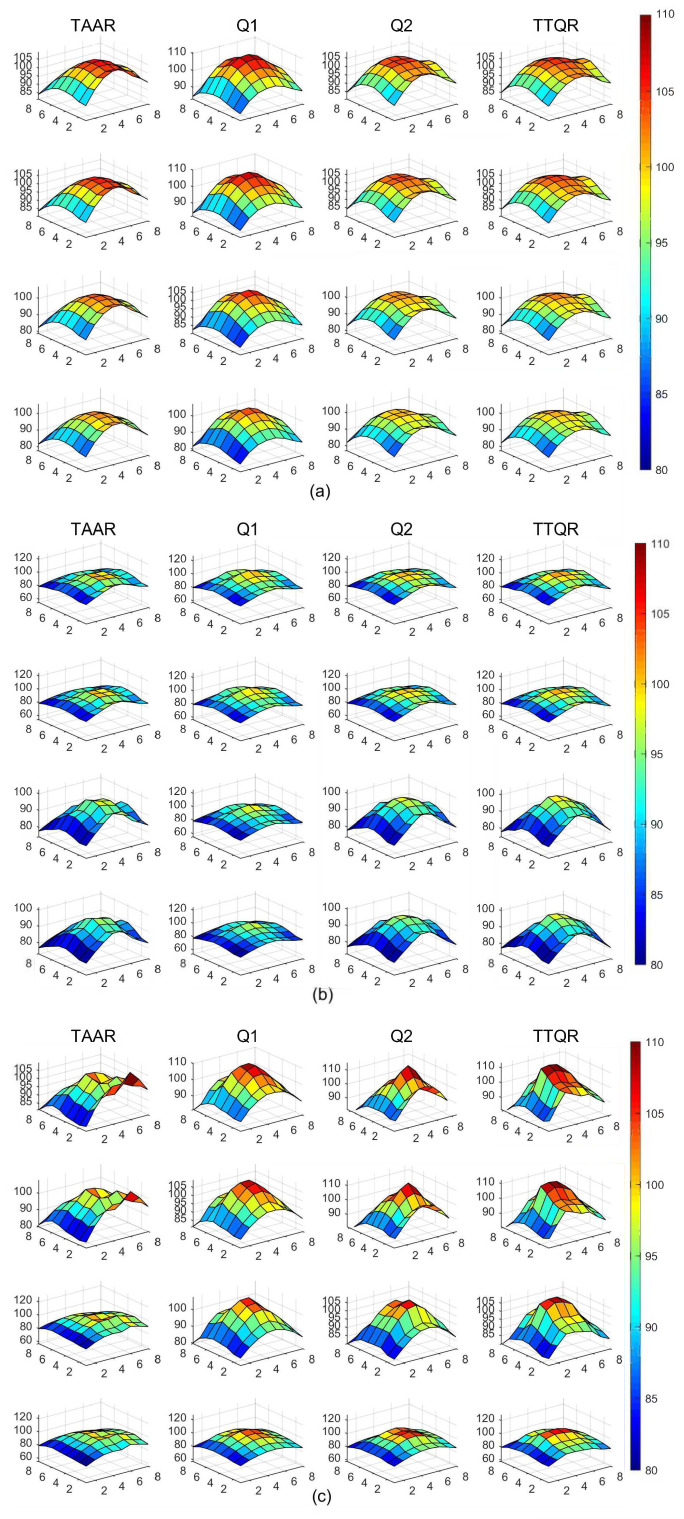
Temperature distribution comparison under different traffic conditions. (**a**) Random. (**b**) Shuffle. (**c**) Bit-reversal.

Figure 5 depicts the 3D plots of the temperature distributions of TTQR, Q1, Q2, and TAAR under the three synthetic flow modes. The averages and standard deviations for the temperature distributions are presented in Table 5. In the proposed TTQR scheme, the average temperature of network nodes is slightly higher than in TAAR, but the difference is within 0.2%, which is almost negligible. TTQR’s network temperature distribution among layers is more uniform than that of the TAAR scheme. This is because TAAR directs packets directly to the layer below when it encounters a throttling node. However, Q-Thermal tracks the temperature of the router in real time, which helps reduce the generation of throttling points. Furthermore, TTQR considers throttling points and global thermal information in routing decisions. Whenever TTQR finds that an area is too hot, it first considers shifting the traffic load to the perimeter link, and then it considers routing to the next layer.

## 6. Conclusions

To eliminate the thermal issue of 3D NoC, previous scholars have proposed many temperature management techniques. However, the problem of performance degradation due to unbalanced traffic remains. In this paper, we have proposed a traffic- and thermal-aware Q-routing algorithm suitable for 3D on-chip networks. The router maintains two Q-tables to store estimates of traffic and temperature information. When making a routing decision, TTQR can choose a low-congestion direction based on the values in the two Q-tables. According to experimental results using synthetic traffic patterns, the performance of the NoC using TTQR routing is significantly better than that of the NoC using TAAR routing. TTQR outperforms the TAAR routing algorithm by an average of 63.6% and 41.4% in average latency and throughput, respectively. This means that the proposed method can achieve higher work efficiency at the same temperature threshold. In addition, because the Q-table of our proposed method is very small and no additional links are required to transfer the learning packets, our hardware overhead is very low. These are significant for the practical application of NoC.

## Figures and Tables

**Table 1:** Q-table configuration for conventional Q-table.

State-Space			Action-Space		Q-Value		Goal
*s*	$s^{\prime }$		*a*		Q(s;a)		*d*
Current-Router	Next-Router		Output-Port		Latency		Destina tion-Router
Node-4	1	3	South	West			Node-0
Node-4	1	-	South	-		-	Node-1
Node-4	1	5	South	East			Node-2
Node-4	3	-	West	-		-	Node-3
Node-4	4	-	Local	-	-	-	Node-4
Node-4	5	-	East	-		-	Node-5
Node-4	3	7	West	North			Node-6
Node-4	7	-	North	-		-	Node-7
Node-4	5	7	East	North			Node-8

**Table 2:** The configuration of Q1-table.

Action	Q1-Value
Output-Port	free_slot_sum
North	A
East	B
South	C
West	D

**Table 3:** The configuration of Q2-table.

Action	Q2-Value
Output-Port	$\mathrm{Avg}\_\mathrm{Temp}{(}^{{}^{\circ}}C)$
North	$45^{{}^{\circ}}$
East	$50^{{}^{\circ}}$
South	$48^{{}^{\circ}}$
West	$67^{{}^{\circ}}$

**Table 4:** Specification of parameters for simulation.

Parameter	Value
Packet size	8 flits
Buffer size	16 flits
Simulation time	$5\times 10^{5}$ cycles
Warm-up time	$1\times 10^{4}$ cycles
Mesh size	8×8×4
Traffic pattern	random, shuffle, bit-reversal
Ambient temperature	25 °C
Routing algorithm	TAAR, TTQR

**Table 5:** Temperature comparison under different conditions for TAAR, Q1, Q2, and TTQR.

	Random				Shuffle				Bit-Reversal			
	TAAR	Q1	Q2	TTQR	TAAR	Q1	Q2	TTQR	TAAR	Q1	Q2	TTQR
Mean(°C)	96.309	96.416	96.564	96.500	89.665	89.934	89.885	89.926	92.610	94.556	94.550	94.337
S.D.(°C)	6.043	6.449	5.421	5.828	5.535	5.552	5.495	5.667	6.616	7.136	7.869	7.375

## Data Availability

The data presented in this study are available on request from corresponding authors.
